# Outcomes of FLEXIBLE Assertive Community Treatment (FACT) Implementation: A Prospective Real Life Study

**DOI:** 10.1007/s10597-015-9831-2

**Published:** 2015-02-04

**Authors:** M. Annet Nugter, Fabiana Engelsbel, Michiel Bähler, René Keet, Remmers van Veldhuizen

**Affiliations:** 1 Department of Research and Monitoring, Mental Health Service Organization GGZ Noord-Holland-Noord, PO Box 18, 1850 BA Heiloo, The Netherlands; 2 Department of Community Mental Health, Mental Health Service Organization GGZ Noord-Holland-Noord, PO Box 18, 1850 BA Heiloo, The Netherlands; 3 Centre for Certification ACT and FACT (CCAF), Praediniussingel 20/9, 9711 AG Groningen, The Netherlands

**Keywords:** FLEXIBLE ACT (FACT), Clinical outcomes, Admissions, Severe mental illness (SMI)

## Abstract

This study aimed to investigate social and clinical outcomes and use of care during and after implementation of FLEXIBLE Assertive Community Treatment (ACT). Three teams and 372 patients were involved. Model fidelity, clinical and social assessments were performed at baseline and after 1 and 2 years. Use of care was registered continuously. Model fidelity was good at the end of the study. Data showed much variation between patients in number and duration of ACT periods. Statistically significant improvements were found in compliance, unmet needs and quality of life. Improvement of quality of life and functioning was related to duration of ACT. The percentage of remissions increased with 9 %. The number of admissions, admission days and face to face contacts differed between ACT and non-ACT patients, but generally decreased. Findings suggest that implementation of FACT results in a more flexible adaptation of care to the needs of the patients.

## Introduction: ACT and FLEXIBLE ACT

This article presents research regarding “FLEXIBLE Assertive Community Treatment”, which is a Dutch version of Assertive Community Treatment (ACT).

Assertive community treatment (Stein and Test 1980) has been established as an effective treatment model for patients with severe mental illness (SMI). The essential ingredients of ACT are: a multidisciplinary team with small, shared caseloads, home based treatment and out of hours availability. In addition, ACT provides integrated dual diagnosis treatment, supports paid employment and includes peer support. The efficacy of ACT has been repeatedly demonstrated in the United States (US). Compared to treatment as usual, ACT results in a reduction of admissions and admission days, more stable housing, greater satisfaction among both patients and families, and less treatment dropout (Marshall and Lockwood 2010). Improvement of psychosocial functioning and the employment situation has not been proven.

Results of ACT in Europe are less convincing. Differences between research findings in the US and European countries were discussed by Burns et al. (Burns et al. 2001). Explanations were sought in differences in the implementation of the model, the organization of the mental health services and in the patient groups. Compared to the US, European researchers paid more attention to the specific features responsible for the effectiveness of ACT than to high program fidelity, in European countries the provision of service is more comprehensive than in the US, and in European studies there are less possibilities to exclude patients who may not benefit much from ACT. More recent, randomized controlled trials in the United Kingdom (UK) and The Netherlands confirm the lack of efficacy of ACT in these countries. The “Randomised evaluation of assertive community treatment (REACT)” (Killaspy et al. 2006, 2009) found no advantage over usual care from community mental health teams in reducing the need for inpatient care and in other clinical outcomes, but participants found ACT more acceptable and engaged better with it. In the Netherlands, ACT was more effective in preventing treatment dropout, but compared to treatment as usual no differences were found with regard to admission days, functioning, psychopathology, quality of life and housing stability (Sytema et al. 2007). Explanations for the lack of results were sought in lack of experience of the teams (Killaspy et al. 2006) and the fact that standard care incorporated many elements that are characteristic of ACT (Killaspy et al. 2006; Sytema et al. 2007). Another Dutch study found a (negative) relationship between level of ACT model fidelity, especially team structure, and some items of the HoNOS and the number of homeless days (Van Vugt et al. 2011). Implementation of fully fledged ACT is especially difficult in rural areas because of the low population density, the lack of adequate services for patients and the lack of personnel (Meyer and Morrissey 2007). This was one of the reasons for the development in the Netherlands of the FLEXIBLE ACT model (Van Veldhuizen 2007; Van Veldhuizen et al. 2008; Van Veldhuizen and Bähler 2013). A FLEXIBLE ACT team supports all patients with a SMI within a catchment area of 50,000 inhabitants, both the 20 % for whom ACT is indicated and the 80 % who would otherwise be served by step-down teams. Like ACT, FLEXIBLE ACT teams are multidisciplinary, including a psychiatrist, case managers, a psychologist, a peer specialist, a supported employment specialist. The teams offer two levels of care: individual case management for most patients, and full ACT when there’s a need for shared caseload and assertive outreach. To combine care for these two groups, the FLEXIBLE ACT team employs a flexible switching system. An important tool that supports this flexibility is the (electronic) FLEXIBLE ACT board (Van Veldhuizen and Bähler 2013). Patients requiring ACT are placed on this electronic board and are discussed daily in the team. For this group, the team adopts a shared caseload approach, a key component of ACT. Patients can be admitted on the board for various reasons: crisis prevention, temporary worsening of symptoms, permanent vulnerability, treatment avoidance, admission to a psychiatric hospital, a court order, or in case a patient is recently registered in the team. For the clients who require less intensive care, the same team provides individual case management with multidisciplinary treatment and support. A FLEXIBLE ACT team shifts from one level to another for longer of shorter periods of time, and patients do not have to be transferred to a different team when their level of needs changes. This ensures continuity of care and that the level of care is finely attuned to the needs of patients. The combination of flexibility and continuity ties in well with the natural course of SMI with its recurring episodes and relapses. There are more than 150 FLEXIBLE ACT teams in the Netherlands and the interest in the model from abroad is growing.

Few studies examined the effects of FLEXIBLE ACT. A prospective Dutch study found a nonsignificant increase in symptomatic remission after the start of FLEXIBLE ACT (Bak et al. 2007); in another Dutch study FLEXIBLE ACT was associated with increased symptomatic remission rates compared to care as usual, but only for patients with an unmet need on psychotic symptoms (Drukker et al. 2008). Two other studies found relatively more outpatient care in FLEXIBLE ACT teams compared to care as usual (Drukker et al. 2011; Drukker et al. 2013); in one of these studies it was found that patients who received FLEXIBLE ACT had relatively high levels of psychosocial functioning (Drukker et al. 2013). Finally, a prospective study in the UK found a reduction in number of admissions and use of beds, which was not offset by crisis home contacts (Firn et al. 2012). In neither of the studies the level of implementation of the FLEXIBLE ACT model has been assessed. The question remains therefore what outcomes may be expected when FLEXIBLE ACT is fully implemented and to what extent fully implemented FLEXIBLE ACT is associated with even better outcomes than the aforementioned studies.

### Purpose of the Study

This study aimed to prospectively follow up three teams that transformed from (partly) intensive case management to FLEXIBLE ACT in order to establish to what extent a fully implemented FLEXIBLE ACT-model enables the improvement of a range of clinical and social outcomes and patient satisfaction with care, while reducing hospital use. To address this aim, level of implementation, clinical and social outcomes, and use of care are repeatedly measured during a period of two and a half years. The outcomes considered are: remission, psychosocial functioning, quality of life, social inclusion, clinical and social needs, patient satisfaction, admissions, admission days, and outpatient contacts.

## Methods

### Patients and Setting

The study was performed from July 2009 to December 2011. 391 patients with SMI of three newly formed FLEXIBLE ACT teams were eligible for this study. The teams were located in a rural area in the Northwest of the Netherlands. Before the transition to FLEXIBLE ACT, these patients were treated by case management teams specialized in the treatment of SMI.

Data are available on the use of care of all 391 patients. However, 19 patients refused to participate, leaving data on the use of care of 372 patients (95.1 %). Clinical outcomes are available of 298 patients (76.2 %). Of 56 of them one or more assessments were missing or incomplete. The data of all 298 patients were used in the analyses. The main reason for nonparticipation was the case managers’ lack of time to complete all assessments within the prescribed time interval of 6 months (see “Procedure” section). The reasons for nonparticipation in the clinical assessments are depicted in Table 1.

**Table 1:** Reasons for nonparticipation clinical assessments

	Frequency	Percentage
All assessments performed	242	61.9
Some assessments missing for practical reasons or patient unable to fill in all forms	56	14.3
Refusal	19	4.9
Lack of time to complete assessments with all patients within time	74	18.9
Total	391	100.0

Table 2 shows the characteristics of the patients who agreed to participate in the study (N = 372) which could be extracted from the electronic files of the patients. Patients for whom all clinical assessments were lacking, more often had no Dutch (either African or Caucasian) origin (Table 2).

**Table 2:** Patient characteristics (N = 372)

	Assessments done (N = 298)	No assessments done (N = 74)^a^
*Primary diagnosis*		
Alcohol or drug abuse or addiction	6 (2.01 %)	0 (0 %)
Anxiety disorders	24 (8.05 %)	4 (5.41 %)
Personality disorders	30 (10.07 %)	7 (9.45 %)
Schizophrenia or other psychotic disorders	180 (60.40 %)	38 (51.35 %)
Depressive disorders	22 (7.38 %)	6 (8.11 %)
Bipolar disorders	23 (7.72 %)	10 (13.51 %)
Other	13 (4.36 %)	9 (12.16 %)
*Sex*		
Male	168 (56.37 %)	38 (51.35 %)
Female	130 (43.62 %)	36 (48.65 %)
*Ethnic background* ^b^		
Dutch–Caucasian	249 (89.89 %)	48 (77.42 %)
Dutch–African	11 (3.97 %)	3 (4.84) %
Other^c^	17 (6.14 %)	11 (17.74 %)
Age: mean and standard deviation	44.12 (12.21)	44.36 (14.28)

^a^Not included in this analysis are patients who refused to participate; no data of these patients are used, including diagnosis and demographics

^b^Of N = 33 patients the ethnicity is unknown

^c^Statistically significant: more nonparticipants were of ‘other’ origin: χ^2^(1) = 9.00, *p* < .01

### Instruments

#### Implementation of FLEXIBLE ACT

The level of implementation of the FLEXIBLE ACT model (fidelity) is measured with the FLEXIBLE ACT-scale (FACTs), developed by the Dutch Centre for Certification ACT and FLEXIBLE ACT (CCAF; www.ccaf.nl). The FACTs consists of 60 items which measure: team structure (12 items), team process (12 items), diagnostics and treatment interventions (13 items), organization of services (10 items), level of social services (five items), use of routine outcome monitoring (ROM, three items), and level of professionalization (five items). All items are scored on a five point rating scale, ranging from 1 to 5, with scoring criteria differing per subscale. The CCAF defines a total score on the FACTs of 3.0 and lower as insufficient; scores 3.1–3.3 indicate that a temporary certificate may be given for 1 year but that improvements are needed to get a final certificate; a temporary certificate is given only once. Scores 3.4–4.0 are sufficient to get the certificate; and scores of 4.1 and higher are regarded as excellent. The FACTs was scored by two independent raters. Their interrater reliability, in terms of the intraclass correlation coefficient, varied from .88 to .99.

Apart from the model fidelity, we retrieved data from the electronic FLEXIBLE ACT boards to compute the number of patients who received ACT, the reason for it, the duration of each ACT-period and the total duration of all ACT-periods.

#### Clinical and Social Outcomes

Remission is assessed with the Remission tool, based on the eight critical remission items of the Positive and Negative Syndrome Scale with which remission is defined (Andreasen et al. 2005; Van Os et al. 2006). In the Remission tool the eight items are dichotomized into score 3 or less and score 4 or more. Score 4 or more indicates that the symptom is influencing the daily life of the patient, while score 3 or less indicate that the symptom is absent or does not influence the patient’s daily life. In case all scores are three or less, a question about the duration of this situation has to be answered. Remission is defined as having no score of 4 or more for a period of 6 months or longer.

Psychosocial functioning was assessed with the Dutch version of the Health of the Nation Outcome Scales (HoNOS; Wing et al. 1998; Mulder et al. 2004). The HoNOS is a 12-item rating scale that measures four subscales: symptoms (three items), disabilities (two items), behavioural problems (three items) and social problems (four items). Items are scored on a 5-point rating scale. The Dutch version of the HoNOS also contains two items about treatment compliance. These two items are used as an extra subscale ‘compliance’. Higher scores indicate poorer functioning.

The Dutch version of the Manchester Short Assessment of Quality of life (MANSA; Priebe et al. 1999) was used to measure quality of life. The MANSA is a self-report rating scale that contains 12 items that are scored on a 7-point rating scale. The MANSA total score was computed, with higher scores indicating better quality of life.

The clinical and social needs of the patients were measured with the Camberwell Assessment of Need Short Appraisal Schedule (CANSAS; Phelan et al. 1995). The CANSAS assesses needs across 22 psychological, social and daily living domains, distinguishing met and unmet needs. The percentage of unmet needs was computed, with higher scores indicating a higher percentage of unmet needs. The percentage was set to zero when patients didn’t have needs.

Social inclusion, in terms of employment status, housing and social contacts, was investigated by using a form that contains 17 questions on: marital status, housing, living situation, income, employment and/or other working activities, education, social contacts, outdoor activities. A social network scale was formed with five items that measure the social network and the number of contacts. Reliability of the scale was moderate (α = .56). Employment status (paid employment or not) and living independent (yes or no) and the social network score were used as outcome variables.

#### Satisfaction with Care

Satisfaction with care was measured with a nationally used Dutch satisfaction self-report questionnaire, that was developed to measure satisfaction and is part of the national set of Performance Indicators in mental health care (www.zichtbarezorg.nl). The 10-point rating scale was used, ranging from 1 (very poor) to 10 (excellent). Patients were asked to indicate their overall satisfaction with care.

#### Patient Characteristics, Hospital Use and Outpatient Contacts

Patient characteristics, data about hospital use and outpatient contacts were retrieved from the electronic patient files. For each period of 6 months we computed the number of patients who were newly admitted, the number of patients who were admitted including the on-going admissions of earlier periods, the total number of hospital days per patient and the total number of outpatient face-to-face contacts..

### Procedure

Two independent raters were trained in the assessment of the FLEXIBLE ACT fidelity with the FACTs. They visited the three teams at the start of the implementation (baseline, T0), and again after one (T1) and two years (T2) of implementation of FLEXIBLE ACT.

The assessments of the clinical outcomes were done by the case managers of the patients. During the first half year the first assessments (T0) were performed. The first assessment of each patient was the starting point for the second (T1) and the third assessments (T2), after twelve and 24 months respectively. The Remission tool, the HoNOS and the CANSAS were scored by means of a structured interview. After the interview the patient was asked to fill in the MANSA and the social inclusion scale. The satisfaction self-report questionnaire was completed separately from the assessment session and could be filled in anonymously and sent to the department of research of the organization. All case managers were trained in the administration and scoring of the instruments that were also used for treatment purposes, as part of the routine outcome monitoring (ROM).

Data on admissions and face-to-face contacts were generated continuously. To study the course of hospital use and outpatient contacts, the study period of two and a half years was divided in five 6 months intervals.

The purpose of the study was explained to the patient. The procedure of engaging permission of the patients to use ROM data and data of the patient’s files was approved by the internal scientific board that reviewed the procedure according to the Dutch law.

### Data Analysis

Patients’ characteristics were compared between participants and nonparticipants with a *t* test for age and χ^2^-tests for ethnicity and diagnoses.

HoNOS, MANSA and CANSAS were regarded as missing when more than 20 % of the items were missing. In case of <20 % missing values, on HoNOS and MANSA a mean score for the remaining items was calculated, multiplied by the number of items and rounded to the nearest number (Downey and King 1998).

Mixed model analyses for repeated measurements were used to analyse clinical outcomes over time. These models explicitly take into account the hierarchical structure of the data, in this study the nesting of repeated measures within persons. In addition, these models are able to handle missing values on one or more assessments, which is common in naturalistic studies.

An unconditional model with two levels (repeated assessments nested within patients) was specified for the HoNOS total score and subscale scores (symptoms, disabilities, behaviour problems and social problems; and the extra compliance scale), for the MANSA total score, the percentage of unmet needs, and the score on the social contacts scale. The factor time and the intercept are used as fixed and random effects. The random intercept, slope and their covariance structure are estimated. A linear growth model was assumed. To analyse the relationship between these outcomes and the total duration of ACT the time by duration of ACT interaction was included into the model.

Generalized mixed model analyses were likewise performed for the dichotomous variables and the variables with a Poisson distribution. With a link function that recognizes the binomial distribution, changes in the number of patients who were admitted, in remission, employed, or living independently were analyzed over the course of 2.5 years. With a link function that recognizes a Poisson distribution the number of admission days and outpatient contacts were analysed. By including a time by duration of ACT interaction we studied the relationship between social outcomes and total duration of ACT. To compare the use of care (contacts and admissions) of patients who had one of more periods of ACT with patients who didn’t, we added a time by group (yes or no ACT) interaction.

For the satisfaction questionnaire descriptive statistics were used, because of the anonymity of this questionnaire.

All analyses were performed with the Statistical Package for the Social Sciences (SPSS), version 20.0.

## Results

### Implementation of FLEXIBLE ACT

#### Model Fidelity

The three teams started with a FACTs score below 3.4, the criterion for certification. Over time, Team 1 and 2 improved gradually to scores of 4.2 and 4.3 at T2, while team 3 ended with a score of 3.6 (Table 3). Analyses of the subscales show that organization of services was good from the beginning. At T2 scores of Team 1 and 2 on the subscales were generally higher than at T0. Team 3 ended with lower scores on two subscales (Table 3).

**Table 3:** Implementation: FACT fidelity scores

FACTS	Team 1			Team 2			Team 3		
	T0	T1	T2	T0	T1	T2	T0	T1	T2
Team structure	3.3	3.6	3.9	3.2	3.7	3.8	3.0	3.6	3.3
Team process	3.2	3.6	4.2	2.7	3.0	4.3	3.1	3.4	3.4
Diagnostics, treatment interventions	2.9	3.4	4.2	2.3	3.5	4.5	3.0	3.4	4.1
Organization of services	4.5	4.3	4.5	4.2	4.2	4.8	3.0	4.4	3.7
Social services	3.8	4.4	4.0	3.8	4.8	4.6	4.0	3.8	4.0
Monitoring	2.3	4.7	4.0	3.0	4.3	5.0	3.5	4.0	3.0
Professionalization	1.6	2.4	4.0	3.2	2.4	4.6	2.8	3.4	2.6
Total score	3.2	3.7	4.2	3.1	3.6	4.3	3.3	3.7	3.6

#### High and Low Intensive Community Care

At the end of each 6 months interval of implementation we counted the number of patients who were placed on the FLEXIBLE ACT board, to get an impression of the proportion of patients who received ACT at one moment. The mean proportion of patients on the FLEXIBLE ACT board on these five moments was 18.3 %; this proportion was rather stable.

During the whole research period of 2.5 years, 240 patients (64.5 %) had been placed on the FLEXIBLE ACT board at least once. Most patients are placed on the FLEXIBLE ACT board once or twice (N = 149, 40.3 %), but there are patients who were placed on the board more than five times (N = 32; 8.6 %).

Table 4 describes the reasons for placement on the FLEXIBLE ACT board. Short-time intensification of care because of a temporary worsening of symptoms, was the most frequent reason (43.0 %), followed by crisis prevention (35.5 %). Detention occurred only once. The duration of ACT varied from 1 day to more than 2 years. The mean duration of ACT was 22.17 weeks (SD = 23.26) and the median was 12.64 weeks.

**Table 4:** Reasons to be placed at the FLEXIBLE ACT board

	Frequency	Percentage
1. Crisis prevention	114	35.5
2. Intensive short-term	160	43.0
3. Intensive long-term	47	12.6
4a. Treatment avoider	9	2.4
4b. High risk treatment avoider	25	6.7
5. Admission	38	10.2
6. Recently registered in the team	26	7.0
7. Detention	1	.3

### Clinical and Social Outcomes

In Table 5 the descriptives of the clinical and social outcomes are depicted. The percentage of patients in remission increased from 33.87 % on T0 to 43.02 % at T2. However, this improvement was not statistically significant. No statistically significant time by duration of ACT was found, indicating that the duration of ACT was not related to changes in the proportion of remission.

**Table 5:** Descriptives of clinical outcomes

	T0	T1	T2
Remission-N (%)	N = 42 (33.87 %)	N = 27 (30.0 %)	N = 37 (43.02 %)
*HoNOS—mean (SD)*			
HoNOS 12 total score	11.80 (6.93)	11.45 (6.44)	10.79 (6.41)
HoNOS symptoms	4.38 (2.59)	4.18 (2.51)	3.96 (2.32)
HoNOS behavior	1.45 (1.78)	1.30 (1.74)	1.18 (1.56)
HoNOS social problems	3.77 (3.32)	3.66 (3.18)	3.54 (3.12)
HoNOS impairments	2.16 (1.80)	2.26 (1.73)	2.08 (1.79)
HoNOS compliance	1.38 (2.04)	1.0 (1.71)	.87 (1.52)
MANSA—Mean (SD)	56.00 (9.34)	57.40 (9.46)	58.04 (9.34)
*CANSAS—Mean* (*SD*)			
Percentage unmet needs	20.51 (21.63)	16.36 (21.03)	16.13 (19.56)
*Social inclusion*			
Social contacts—Mean (SD)	23.33 (7.39)	23.75 (7.10)	24.08 (6.86)
Paid employment—N (%)	N = 34 (11.93 %)	N = 23 (11.22 %)	N = 26 (14.13 %)
Living independently—N (%)	N = 263 (91.63 %)	N = 196 (92.89 %)	N = 188 (93.53 %)

No significant changes were found on the HoNOS total score and its four subscale scores. Improvement on the compliance subscale of the Dutch version was statistically significant: mean scores on this scale decreased statistically significant (*β* = −.22, *t* (215.549) = −3.13, *p* < .01) from 1.38 (SD = 2.04) to .87 (SD = 1.52) (Table 4). A statistically significant time by duration of ACT interaction effect was found for the HoNOS total score (*β* = .005, *t* (233.888) = 3.43, *p* < .01), and for scores on symptoms (*β* = .002, *t*(257.425) = 3.76, *p* < .001), social problems (*β* = .002, *t*(243.190) = 2.62, *p* < .01) and compliance (*β* = .001, t(236.482) = 2.50, p < .05), indicating that the patients who remained on the board for longer periods showed less improvement in functioning.

Total score on the MANSA improved statistically significant from a mean of 56.00 (SD = 9.34) at T0 to 58.04 (SD = 9.34) at T2 (*β* = 1.03 *t*(220.229) = 3.28, *p* < .01). In addition, a statistically significant time by duration of ACT interaction effect was found (β = −.006, t(243.051) = −2.48, *p* < .05), indicating that the patients who remained on the board for longer periods showed less improvement in quality of life.

The mean percentage of unmet needs decreased statistically significantly from 20.51 % (SD = 21.63) at T0 to 16.13 % (SD = 19.56) at T2 (*β* = −2.24, *t*(243.048) = −2.72, *p* < .01).

Few patients had a paid job both at the beginning and the end of the study, 11.9 and 14.1 % respectively. Most patients lived independently, both at the beginning and the end of the study, 91.6 and 93.5 % respectively. Scores on the social contact scale did not change significantly (Table 5). A statistically significant time by duration of ACT interaction effect was found with regard to the patient’s living situation (β = −.007, t(695) = −2.44, *p* < .05), indicating that the patients who didn’t live independently remained on the board for longer intervals.

### Use of Care

#### Termination of Treatment

During the 30 months, the treatment of 44 patients was terminated. Because of this small number, further statistical analyses weren’t performed. In most instances (N = 22) treatment was terminated because psychiatrist and patient decided that treatment wasn’t necessary anymore. Other reasons were: moving to another part of the country (N = 7), death, no suicide (N = 5), suicide (N = 3), other or unclear (N = 7). No patient dropped out of treatment.

#### Admissions and Outpatient Contacts

The proportion of patients who were admitted reduced statistically significant from 14.0 % in the first period of 6 months to 8.6 % in the last period of 6 months (*β* = −.24, *t*(1.858) = −2.74, *p* < .01). In addition, we found a statistically significant main effect for group (ACT or not), (*β* = −2.10, *t*(1.856) = −2.24, *p* < .05). We didn’t find a statistically significant time by ACT (yes or no) interaction effect. In the ACT group, the proportion of admissions reduced from 19.6 % in the first 6 months to 13.3 % in the last 6 months; in the non-ACT group the proportion admission reduced from 3.8 to 0 %.

With regard to the proportion of new admissions, again we found a statistically significant reduction in admissions (*β* = −.25, *t*(1.858) = −3.36; *p* < .01), and a statistically significant main effect for group (*β* = −1.82, *t*(1.856) = −2.24, *p* < .05), indicating more admissions among the ACT group. No statistically significant time by group interaction effect was found.

With regard to the proportion of admissions and the proportions of new admissions, the main effect for ACT (yes or no) became nonsignificant when we omitted the patients who ended the treatment before the end of the study. Other results were comparable.

**Fig. 1:**
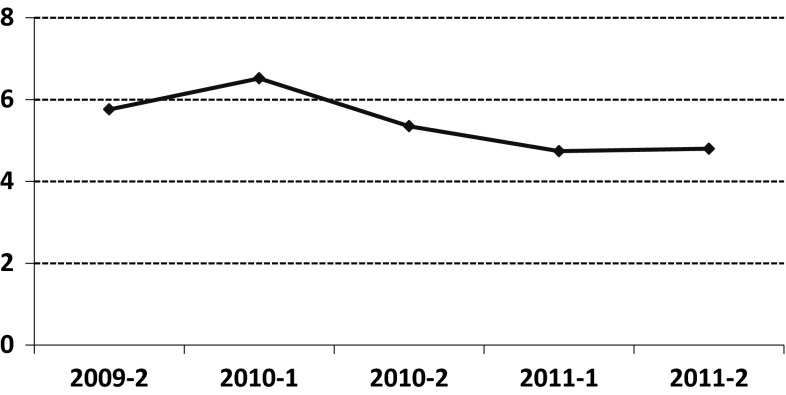
Mean number of hospital days per 6 months interval

The mean number of hospital days per patient decreased statistically significant from 5.8 (SD = 21.23) to 4.8 (SD = 20.21) (*β* = −.15, *t*(1.858) = −2.60, *p* < .01). As can be seen in Fig. 1 the reduction was preceded by an initial increase. A statistically significant main effect for group indicated a higher mean number of hospital days in the ACT group compared to the non-ACT group (*β* = 1.88, *t*(1.856) = 2.55, *p* < .05). No significant time by group interaction effect was found, indicating no clear difference in reduction in both groups.

Comparable results with regard to the mean number of hospital days were found when we omitted the patients who ended their treatment before the end of the study.

The mean number of outpatient contacts reduced from 18.98 (SD = 16.05) in the first half year to 18.09 in the last 23.69. This reduction is statistically significant (*β* = −.08, *t*(1.858) = −5.98, *p* < .001). Like the number of hospital days, the reduction in contacts was preceded by an initial increase. Further analyses showed both a statistically significant main effect for group (*β* = .39, *t*(1.856) = 3.91, *p* < .001) and a statistically significant time by group interaction effect (*β* = .067, *t*(1.856) = 2.33, *p* < .05). The mean number of outpatient contacts of the ACT group increased somewhat from 21.09 (SD = 16.67) in the first 6 months to 21.46 (SD = 26.57) in the last 6 months, while the mean number of contacts in the non-ACT group reduced from 15.16 (SD = 14.13) to 11.95 (SD = 15.58). Comparable results were found when we omitted the group that ended treatment before the end of the research period, except for the interaction effect.

### Patient’s Satisfaction

The number of patients who filled in the satisfaction questionnaire reduced from N = 226 in the first half year to N = 106 in the third. This makes comparison difficult. The mean satisfaction increased from 7.53 (SD = 1.32) to 7.69 (SD = .84).

## Discussion

This study aimed to establish both clinical and social outcomes and outcomes in terms of use of care during and after the implementation of the FLEXIBLE ACT model. It is the first study in which the implementation of FLEXIBLE ACT was repeatedly measured with the FLEXIBLE ACT fidelity scale and in which data about the flexible intensification of contact were taken into account. An important finding is that implementation of FLEXIBLE ACT takes time, at least a year, and that rate of implementation may differ between teams. The teams that were involved in the study already had experience with intensive case management of patients with SMI, which is reflected in the total scores on the FACTs at baseline, that were all higher than 3.1. According to accreditation guidelines, this score leads to a temporary certificate, with recommendations for improvements within a year. After 2 years of implementation two of the three teams had excellent fidelity scores on the FACTs total scores and many of its subscores. This is rather unusual, and may be the result of the fact that these teams worked in the same building and the fact that the psychiatrists of one of these teams was very experienced in FLEXIBLE ACT as well as in implementation in general. The third team, that scored somewhat lower, operated in a different area and more independently. It should be emphasized that a total score of 3.6 or 3.7 is a very common score for certified FLEXIBLE ACT teams. The lower scores of this team on professionalization and monitoring are fed back to the team as aspects that may be improved but not essential enough to prevent certification. Unfortunately we were unable to relate the implementation scores directly to the outcomes, like Van Vugt et al. (2011) did for ACT, because we did not have enough teams with enough variation in model fidelity scores.

Data from the electronic FLEXIBLE ACT board show the flexibility of the model: although at any moment about 18 % of the patients received ACT, during the whole period almost two-thirds of the patients had one or more periods in which the care was intensified, and there were huge differences in both the number of periods of intensification and the duration of intensification. Often patients were admitted on the board to prevent crisis or because symptoms worsened temporarily. These data reflect that the application of the FLEXIBLE ACT model results in a carefully monitoring of the condition and situation of the patients and a flexible adaptation of care to patients’ needs, without discontinuation of care.

The results show a decrease both in the number of patients who were admitted as well as in the number of hospital days. This is in agreement with the study of Firn et al. (2012) study, who found a decrease in the percentage of patients who were admitted from 38 to 22 % in the first year after the introduction of FLEXIBLE ACT. In this study the percentage of patients who were admitted was much lower both at the start and at the end of the study. Still we found a decrease of 5.4 %. Although the proportion of admissions and number of admission days were lower in the ACT group compared to the group of patients who didn’t receive ACT, the reduction in admission and admission days didn’t differ significantly.

Like Firn et al., we also found a slight, but statistically significant decrease in the mean number of outpatient face-to-face contacts. However, the mean number of face to face contacts in this study was much lower than the 60 contacts per year that resulted after the introduction of FLEXIBLE ACT in the study of Firn et al. (2012). On a comparable yearly basis the mean number of face to face contacts in this study reduced from 36 to 32. However, further analyses showed a clear decrease in number of contacts for the patient who didn’t receive ACT, ending in a much lower mean of 24 contacts on an annual basis. For the patients who received ACT the number of face-to-face contacts not only was clearly higher (about 43 on a yearly basis), but didn’t change much. The large standard deviation showed much variability in the number of contacts.

In discussion with the teams it was hypothesized that, in the beginning, teams operated cautiously with regard to fully fledged application of FLEXIBLE ACT, which may explain the increase in both the number of hospital days and outpatient face-to-face contacts during the first period of implementation. The further reduction in hospitals days and contacts may reflect the flexibility of FLEXIBLE ACT to adapt the intensity of care, with the result that no more care is offered than needed.

Outcomes in terms of needs for care and quality of life are positive: the proportion of unmet needs decreased from 20 to 16 %, which may be another indication that FLEXIBLE ACT is indeed successful in adapting the care to the needs of the patients. In addition, the patients reported a better subjectively experienced quality of life, which is in agreement with the fact that there is an inverse relationship between needs for care and subjectively experienced quality of life: higher levels of quality of life go hand in hand with fewer unmet needs for care (Björkman and Svensson 2005). Psychosocial functioning, social inclusion and satisfaction with care did not change during the 2 years of implementation of FLEXIBLE ACT. The percentage of patients with paid employment remained low. For housing a further improvement wasn’t expected since the percentage of patients who lived independently was already rather high at the start of the study. Compared to the Dutch study of Bak et al. (2007), who based their definition of remission only on the symptom scores and did not include the duration criterion, the percentage of patients with psychotic symptoms who were in remission was rather high at the start of the study: 34 %. Still, we found an increase in the remission rate of 9 %.

Further analyses showed there to be an interaction between duration of ACT and improvement of psychosocial functioning (symptoms and social problems), quality of life and compliance. The results indicate that patients who do not improve need longer periods of ACT. Comparable results were found by Kortijk et al. (2012), who found a negative correlation between duration of ACT and change in HoNOS total scores. They concluded that patients in ACT teams with different treatment durations constitute distinguishable groups with different outcomes. Further research may be undertaken to see if this applies to FLEXIBLE ACT as well. An additional interaction effect was found with regard to patient’s living situation, indicating longer periods of ACT for patients who don’t live independently.

There were no dropouts and treatment compliance increased significantly, which may be the result of the continuity of care that FLEXIBLE ACT teams offer.

A limitation of the study concerns the number of teams involved. Because we have data of only three teams we could not conduct a direct analysis of the relation between level of fidelity and outcomes.

An important weakness of this study is its naturalistic character and uncontrolled conditions. We only had data from the moment we started with the implementation. Effects of the implementation couldn’t be compared to a former situation. Neither did we have data of teams that hadn’t implemented FLEXIBLE ACT yet. So we are not sure to what extent the result may be ascribed to the implementation of FLEXIBLE ACT. An alternative explanation of course is that both case managers and patients were not blind to the fact that FLEXIBLE ACT was being implemented which may have influenced their way of reporting. In addition, the attention given to the performance of the teams may have contributed to the improvement of outcomes. On the other hand, the fact that fidelity scores improved showed that teams were functioning increasingly following the FLEXIBLE ACT model.

Because of the lack of a control group, we cannot rule out that the results may be ascribed to regression to the mean. However, the teams with which we started already functioned rather well: before the transition to FLEXIBLE ACT, treatment was provided by (partly) intensive case management teams specialized in SMI (Dekker et al. 2000). On the FACTs the newly formed FLEXIBLE ACT teams scored almost at the level of the criterion for certification. Also, the percentage of remissions was rather high, while the number of admissions, the mean number of hospital days, the mean number of face to face contacts were already rather low, which makes it unlikely that regression to the mean is the only explanation.

In addition, data from the electronic database and the clinical outcomes complement each other and are in line with what we expect when implementing FLEXIBLE ACT: a shared caseload and flexibility in change from a more intensive to a lesser intensive level should lead to more efficiency in the use of care and better adaptation to the needs of the patients.

Complete clinical outcomes were only available for 62 % of the patients, for another 14 % assessments were not complete; of the remaining 24 % we didn’t have any data. Fortunately, we were able to use all data, also data of the patients for whom assessments were missing. However, response analyses showed that the patients of whom we had at least one assessment more often had the Dutch nationality, indicating a restriction of the generalizability of the results.

It may be concluded that implementation of FLEXIBLE ACT takes time, even when teams already have experience with intensive case management. FLEXIBLE ACT is associated with a greater subjective quality of life, a reduction in the percentage of unmet needs and an increase in compliance. At the same time, there is less hospital use and a decrease in the number of outpatient contacts. These positive results apply to the ACT group as well as to the group that didn’t need ACT. The strength of FLEXIBLE ACT is it’s flexible adaptation of care to patient’s needs, without discontinuation of care. This applies not only to ACT patients, but to the whole group of patients with SMI.

Further research into the effectiveness of FLEXIBLE ACT should include both control groups and measures for the level of implementation of the model. In addition, it would be worthwhile to explore the relationship between the extent to which a FLEXIBLE ACT team pays attention to and undertakes activities directed at the patient’s working situation and social relationships in order to find explanations for the lack of effectiveness with regard to these outcomes.

## References

[CR1] Andreasen NC, Carpenter WT, Kane JM, Lasser RA, Marder SR, Weinberger DR (2005). Remission in Schizophrenia: Proposed criteria and rationale for consensus. American Journal of Psychiatry.

[CR2] Bak, M., van Os, J., Delespaul, P., de Bie, A., á Campo, J., Poddighe, G., & Drukker, M. (2007). An observational, “real life” trial of the introduction of assertive community treatment in a geographically defined area using clinical rather than service use outcome criteria. *Social Psychiatry and Psychiatric Epidemiology,**42*, 125–130. 10.1007/s00127-006-0147-y 17235445

[CR3] Björkman T, Svensson B (2005). Quality of life in people with severe mental illness. Reliability and validity of the Manchester Short Assessment of Quality of Life (MANSA). Nordic Journal of Psychiatry.

[CR4] Burns T, Fioritti A, Holloway F, Malm U, Rössler W (2001). Case management and assertive community treatment in europe. Psychiatric Services.

[CR5] Dekker J, Kluiter H, Kroon H, Polstra L (2000). Community care arrangements in the Netherlands. European Journal of Psychiatry.

[CR6] Downey RG, King CV (1998). Missing data in Likert rating: A comparison of replacement methods. The Journal of General Psychology.

[CR7] Drukker M, Maarschalkerweerd M, Bak M, Driessen G, A Campo J, De Bie A, Poddighe G, Van Os J, Delespaul Ph (2008). A real-life observational study of the effectiveness of FACT in a Dutch mental health region. BMC Psychiatry.

[CR8] Drukker M, Van Os J, Sytema S, Driessen G, Visser E, Delespaul Ph (2011). Function assertive community treatment (FACT) and psychiatric service use in patients diagnosed with severe mental illness. (2011). Epidemiology and Psychiatric Science.

[CR9] Drukker M, Visser E, Sytema S, Van Os J (2013). Flexible assertive community treatment: Severity of symptoms and psychiatric health service use; a real life observational study. Clinical Practice & Epidemiology in Mental Health.

[CR10] Firn M, Hindhaugh K, Hubbeling D, Davies G, Jones B, White SJ (2012). A dismantling study of assertive outreach services: Comparing activity and outcomes following replacement with the FACT model. Social Psychiatry and Psychiatric Epidemiology.

[CR11] Killaspy H, Bebbington P, Blizard R, Johnson S, Nolan F, Pilling S, King M (2006). The REACT study: Randomised evaluation of assertive community treatment in north London. British Medical Journal.

[CR12] Killaspy H, Kingett S, Bebbington P, Blizard R, Johnson S, Nolan F, Pilling S, King M (2009). Randomised evaluation of assertive community treatment: 3-Year outcomes. British Journal of Psychiatry.

[CR13] Kortrijk HE, Mulder CL, Drukker M, Wiersma D, Duivenvoorden HJ (2012). Duration of assertive community treatment and the interpretation of routine outcome data. Australian and New Zealand Journal of Psychiatry.

[CR14] Marshall M, Lockwood A (2010). Assertive community treatment for people with severe mental disorders. Cochrane database of systematic review.

[CR15] Meyer PS, Morrissey JP (2007). A comparison of assertive community treatment and intensive case management for patients in rural areas. Psychiatric Services.

[CR16] Mulder CL, Staring ABP, Loos J, Buwalda VJA, Kuijpers D, Sytema S, Wiersma AI (2004). De Health of the Nation Outcome Scales (honos) als instrument voor ‘routine outcome assessment’. Tijdschrift voor Psychiatrie.

[CR17] Phelan M, Slade M, Thornicroft G, Dunn G, Holloway F, Wykes T, Strathdee G, Loftus L, McCrone P, Hayward P (1995). The Camberwell assessment of need: The validity and reliability of an instrument to assess the needs of people with severe mental illness. British Journal of Psychiatry.

[CR18] Priebe S, Huxley P, Knight S, Evans S (1999). Application and results of the Manchester Short Assessment of Quality of Life (MANSA). International Journal of Social Psychiatry.

[CR19] Stein LI, Test MA (1980). Alternatives to mental hospital treatment Part I: Conceptual model treatment program and clinical evaluation. Archives of General Psychiatry.

[CR20] Sytema S, Wunderink L, Bloemers W, Roorda L, Wiersma D (2007). Assertive community treatment in the Netherlands: A randomized controlled trial. Acta Psychiatrica Scandinavica.

[CR21] Van Os J, Burns T, Cavallaro R, Leucht S, Peuskens J, Helldin L, Bernardo M, Arango C, Fleischhacker W, Lachaux B, Kane JM (2006). Standardized remission criteria in schizophrenia. Acta Psychiatr Scandinavica.

[CR22] Van Veldhuizen JR (2007). FACT: A Dutch version of ACT. Community Mental Health Journal.

[CR23] Van Veldhuizen, J. R., & Bähler, M. (2013). *Manual flexible ACT, vision, model, practice and organization*. www.factfacts.nl

[CR24] Van Veldhuizen, J. R., Bähler, M., Polhuis, D., & van Os, J. (2008). *Handboek FACT*. De Tijdstroom Utrecht.

[CR25] Van Vugt MD, Kroon H, Delespaul PAEG, Dreef FG, Nugter A, Roosenschoon BJ, Van Weeghel J, Zoeteman JB, Mulder CL (2011). Assertive community treatment in the Netherlands: Outcome and model fidelity. Canadian Journal of Psychiatry.

[CR26] Wing JK, Beevor AS, Curtis RH, Park SBG, Haddem S, Burns A (1998). Health of the nation outcome scales: Research and development. British Journal of Psychiatry.

